# Age at first drink below 19 years and all-cause mortality in Chinese older adults: a 10-year prospective cohort study

**DOI:** 10.3389/fnut.2026.1873121

**Published:** 2026-07-30

**Authors:** Shipeng Zhang, Yuying Guan, Yanjie Jiang, Kun Zhu, Hanyu Wang, Xingyi He, Qinxiu Zhang, Yan Lu, Li Fu, Li Zhou

**Affiliations:** 1 Hospital of Chengdu University of Traditional Chinese Medicine, Chengdu, China; 2 Nanjing University of Chinese Medicine, Nanjing, China; 3 Guangzhou University of Chinese Medicine, Guangzhou, China; 4 Hospital of Chengdu University of Traditional Chinese Medicine, Guangzhou, China

**Keywords:** AFD, cause mortality, cohort, HR, older adults

## Abstract

**Background:**

The association between age at first drink (AFD) and long-term survival in older adults remains unclear, especially in Chinese populations with high alcohol metabolism genetic variants. We aimed to investigate whether AFD < 19 years (legal drinking age in China) was associated with all-cause mortality in Chinese older adults.

**Methods:**

This prospective cohort study included 7,187 participants aged ≥65 years from the Chinese Longitudinal Healthy Longevity Survey (2008–2018). AFD was categorized as never drinking, early drinking (AFD < 19 years), and non-early drinking (AFD ≥19 years). The primary outcome was all-cause mortality. Cox proportional hazards models were used, with progressive adjustment for sociodemographic, lifestyle, dietary (hPDI), BMI, and chronic diseases. Dose–response analysis within early drinkers used ≤ 1 cup/day as the reference category.

**Results:**

During the 10-year follow-up period, early drinking was associated with a 16% higher risk of all-cause mortality (HR = 1.16, 95% CI: 1.01–1.32) compared with never drinking, while non-early drinking showed no significant association. Among early drinkers, a dose–response relationship was observed: ≥3 cups/day increased mortality risk by 38% (HR = 1.38, 95% CI: 1.06–1.79, *p* for trend = 0.015). The association was stronger in participants aged < 75 years (HR = 1.99), those with heart disease (HR = 1.63), dementia (HR = 1.91), or married and living with spouse (HR = 1.60). Sensitivity analyses (excluding chronic diseases or early deaths, propensity score matching) confirmed the robustness.

**Conclusions:**

AFD below 19 years was significantly associated with higher all-cause mortality in Chinese older adults, especially in younger-old individuals and those with heart disease, dementia, or married status.

## 1 Introduction

Alcohol consumption is a leading risk factor for the global disease burden and premature mortality. An estimated 3 million people worldwide die annually from alcohol-related causes, accounting for 4.7% of all deaths (1). Long-term excessive drinking substantially increases the risks of liver cirrhosis, various cancers (e.g., oral, esophageal, liver, and colorectal), cardiovascular diseases (particularly hemorrhagic stroke), and injury-related deaths (2–5). Although numerous studies have demonstrated the wide-ranging harms of alcohol to health, evidence remains insufficient regarding the impact of the timing of alcohol exposure—specifically, the age at first drink (AFD)—on the risk of long-term mortality.Adolescence and young adulthood are critical windows for brain development. Initiating alcohol use during this period is closely associated with later more severe drinking patterns, alcohol use disorders, and various health risk behaviors (6, 7). Previous studies have suggested that earlier AFD may independently increase the risk of adverse health outcomes in adulthood, independent of lifetime alcohol consumption (8, 9). For instance, a 27-year follow-up study of a U.S. community-based population found that individuals who experienced their first intoxication before age 15 had a significantly higher risk of all-cause mortality (8); similarly, a Finnish birth cohort study reported that early alcohol consumption (< 18 years) was associated with an increased risk of premature death before age 30 (10). However, most of these studies focused on younger and early middle-aged populations and were predominantly conducted in high-income Western countries. The association between AFD and long-term survival outcomes in older adults—particularly among Chinese older adults—remains unclear.

China is one of the world's largest alcohol markets. In recent years, alcohol consumption has continued to rise, and population aging has accelerated (11). In Chinese culture, drinking is often closely intertwined with social and workplace activities, rendering early drinking more prevalent (12). However, no study based on Chinese older adult cohorts has systematically assessed the long-term impact of AFD (e.g., whether it occurs before the legal drinking age) on all-cause mortality. Given that the Chinese population's sensitivity to alcohol toxicity may differ from that of other ethnic groups (13), targeted research in this population is warranted.

Against this background, this study used the Chinese Longitudinal Healthy Longevity Survey (CLHLS) to investigate the association between early alcohol consumption (first drinking age < 19 years, the legal drinking age in China) and all-cause mortality. We hypothesized that, compared with never drinkers, early drinkers would have a significantly higher risk of all-cause mortality. Furthermore, we explored the modifying effects of factors such as sex and current alcohol consumption volume on this association. Thus, this study provides epidemiological evidence to inform targeted alcohol intervention strategies (e.g., delaying drinking initiation) for older adults.

## 2 Methods

### 2.1 Research design and data sources

This study is based on the survey data from CLHLS conducted from 2008 to 2018. CLHLS is a nationwide prospective cohort study covering 23 provinces in China. It employs a multi-stage stratified cluster random sampling method and the survey subjects are community-dwelling elderly individuals aged 65 and above. This study has been approved by the Ethics Committee of Peking University (Approval Number: IRB00001052-13,074), and all participants or their legal representatives have signed informed consent forms (14). This study has been reported in accordance with the STROBE guidelines.

### 2.2 Study population

This study included participants who had complete baseline data and were aged 65 or above in the 2008 survey. Exclusion criteria were as follows: (1) Those with missing information on drinking status at baseline; (2) Those with missing or uncalculable daily alcohol consumption data; (3) Individuals with more than 5% missing values for key covariates (such as age, sex, smoking status, exercise, dietary data, etc.); (4) Individuals who did not undergo follow-up after 2008.

### 2.3 Exposure assessment

#### 2.3.1 Classification of drinking status

Based on the 2008 baseline data, we classified participants' drinking status using three questions: (1) “Did you drink in the past?” (2) “Do you currently drink?” and (3)“At what age did you start drinking?”

- Never drinking group (No drinking): participants who answered “No” to both the first and second questions.
- Early drinking group (Early drinking): participants who answered “Yes” to the first question and reported an age at drinking initiation younger than 19 years.
- Non early drinking group (Non-early drinking): participants who answered “Yes” to the first question and reported an age at drinking initiation of 19 years or older.

#### 2.3.2 Alcohol intake

For drinkers, the average daily alcohol intake was calculated based on the question: “How much alcohol do you consume on average every day?” The response unit was the participant's self-reported daily drinking unit (referred to as “cup”), which reflects the participant's usual drinking vessel (e.g., a small cup or glass) in daily life in China. This unit should not be interpreted as an eight-fluid-ounce measuring cup or an internationally standardized drink. Participants who reported alcohol use but consumed less than 1 cup per day on average (e.g., drinking every other day) were retained as drinkers and categorized into the lowest intake group. According to the distribution and clinical significance, the daily alcohol intake was divided into: ≤ 1 cup, 2 cups, and ≥3 cups. In the dose-response analysis, the dose was included as a categorical variable with the ≤ 1 cup per day group serving as the reference category. Because the CLHLS dataset did not consistently provide information on beverage type (e.g., baijiu, beer, wine) or alcohol concentration, we could not convert the reported intake into grams of pure ethanol or other standardized metrics. This limitation should be considered when interpreting the dose-response findings.

### 2.4 Outcome variables

The primary outcome was all-cause mortality. Death information was obtained through the death registration data linked from the CLHLS, and the follow-up cut-off date was December 31, 2018. Survival time was defined as the interval (in years) from the baseline survey date to the date of death or the last follow-up date.

### 2.5 Covariates

Based on previous literature and causal diagrams, we selected the following covariates for correction.

Demographic variables: age (continuous), sex (male/female), Ethnicity (han/other), marital status (Living with spouse in marriage/divorced/unmarried/separated /widowed), education level (educated/not educated), personal annual income (continuous), occupation.

Lifestyle variables: smoking status (Yes/No), Exercised (“Whether exercising regularly”, Yes/No), sleep quality (good/so so/bad/very bad/very good), sleep duration (hours/day, continuous).

Body size indicators: body mass index (BMI, categorized: < 25 kg/m^2^, 25–29.9 kg/m^2^, >29.9 kg/m^2^).

Diet quality: healthy plant-based diet index (hPDI), calculated based on the CLHLS food frequency questionnaire, with higher scores indicating healthier dietary quality (14).

Baseline chronic disease history: hypertension, diabetes, heart disease, cerebrovascular disease (CVD), cancer, dementia (all are yes/no).

Covariates were selected a priori based on previous epidemiological evidence, biological plausibility, and causal considerations. Demographic and socioeconomic variables, including age, sex, ethnicity, marital status, education, income, and occupation, were included because they may influence both the timing of drinking initiation and long-term mortality risk. Lifestyle-related factors, including smoking status, regular exercise, sleep quality, and sleep duration, were adjusted for because they are closely associated with alcohol-related behaviors and survival in older adults. BMI and dietary quality, assessed using the healthy plant-based diet index (hPDI), were included to account for differences in nutritional and metabolic status. Baseline chronic diseases, including hypertension, diabetes, heart disease, cerebrovascular disease, cancer, and dementia, were further adjusted for in the fully adjusted model because these conditions are strong predictors of mortality and may also be related to alcohol exposure history. We anticipated that the association between early drinking and mortality would be attenuated but remain observable after adjustment for these factors, and that stronger associations might be present among participants with higher alcohol intake or pre-existing chronic conditions.

### 2.6 Statistical analysis

The statistical methods were chosen according to the prospective cohort design, the time-to-event nature of the outcome, and the distribution of variables. Baseline characteristics were summarized using means with standard deviations or medians with interquartile ranges (IQRs) for continuous variables and frequencies with percentages for categorical variables. Non-parametric tests were used for continuous variables when distributional assumptions were not met, and chi-squared or Fisher's exact tests were used for categorical variables according to expected cell counts. Cox proportional hazards regression was selected as the primary analytical approach because it allows estimation of hazard ratios for all-cause mortality while accounting for varying follow-up time and censoring. Sequential models were constructed to evaluate the stability of the association after progressive adjustment for sociodemographic, lifestyle, dietary, anthropometric, and clinical factors. The proportional hazards assumption was assessed using Schoenfeld residuals. Dose-response analysis was performed to examine whether greater daily alcohol intake among early drinkers was associated with progressively higher mortality risk. Subgroup and interaction analyses were conducted to explore potential effect modification by demographic, lifestyle, and clinical factors. Sensitivity analyses, including exclusion of participants with baseline chronic diseases, exclusion of early deaths, and propensity score matching, were used to evaluate the robustness of the findings and to reduce concerns regarding reverse causality, baseline morbidity, and covariate imbalance.

#### 2.6.1 Baseline characteristics

Continuous variables were summarized as mean ± standard deviation (SD) or median with interquartile range (IQR), as appropriate, and categorical variables were summarized as frequencies and percentages. Group comparisons for continuous variables were performed using the Wilcoxon rank-sum test or Kruskal–Wallis test. For comparison between groups of categorical data, we used the Fisher exact test for expected frequencies < 5; otherwise, we used the Chi-squared test.

#### 2.6.2 Cox proportional hazards regression model

The Cox proportional hazards regression model was used to assess the association between drinking types and the risk of all-cause mortality. Three successive models were constructed:

Model 1: No adjustment was made for any covariates (the crude model).

Model 2: Adjustments were made for sociodemographic variables, lifestyle variables, BMI, occupation, and hPDI.

Model 3: In Model 2, all baseline chronic diseases (hypertension, diabetes, heart disease, CVD, cancer, dementia) were further adjusted.

The proportional hazards assumption was tested using the Schoenfeld residuals, and no significant deviation was found (*p* > 0.05).

#### 2.6.3 Dose-response analysis

For early drinkers, the daily alcohol consumption (1, 2, ≥ 3 drinks) was used as the categorical variable. By using the participants who consumed 1 drink daily in the early drinking group as the reference group, three Cox models were employed to assess the association between dose and the risk of all-cause mortality. At the same time, the trend *p* value (*p* for trend) was calculated, and the dose was included as a continuous variable (with median assignment) in the model for linear trend testing.

#### 2.6.4 Subgroup analysis

To assess the robustness of the results and potential modifiers of the effects, subgroup analysis was conducted based on the following variables: sex, BMI classification, hypertension, diabetes, heart disease, CVD, cancer, dementia, smoking status, occupation, physical activity, sleep quality, ethnicity, marital status, and educational level. A adjusted model was constructed for each subgroup to estimate the HR. The *p* value for interaction was calculated by introducing the product term of the type of alcohol consumption and the subgroup variables.

#### 2.6.5 Sensitivity analysis

We conducted three sensitivity analyses to test the robustness of the main results:

Excluding baseline patients with chronic diseases: remove participants who had hypertension, diabetes, heart disease, CVD, cancer, or dementia at baseline, and use the remaining life-related covariates as confounding factors to re-perform the Cox regression analysis.

Excluding early deaths: remove participants who died within the first 2 years of follow-up to reduce reverse causality bias (due to reduced dosage or alcohol cessation caused by underlying diseases).

Propensity score matching (PSM): treat early alcohol consumption as the treatment group and no alcohol consumption as the control group. Use the nearest neighbor matching method (1:1) to match age, sex, BMI, and smoking status, with a cutoff value of 0.02. After matching, evaluate the standardized difference (SMD) of covariates, requiring all SMDs to be < 0.1. After matching, three statistical models were constructed in the sample to explore the association between EG and all-cause mortality, to verify the stability of the main analysis.

To assess the potential influence of missing alcohol-exposure data and complete-case selection bias, we additionally performed a multiple imputation sensitivity analysis for missing alcohol-exposure information using baseline demographic, socioeconomic, lifestyle, clinical variables, and survival outcome information. The Cox proportional hazards model was then repeated in the imputed dataset using the same covariate adjustment strategy as the main analysis.

All statistical analyses were completed using the “survival” package and “MatchIt” package in R software (version 4.2.1). Two-sided test *p* < 0.05 was considered statistically significant.

## 3 Result

**Figure 1:**
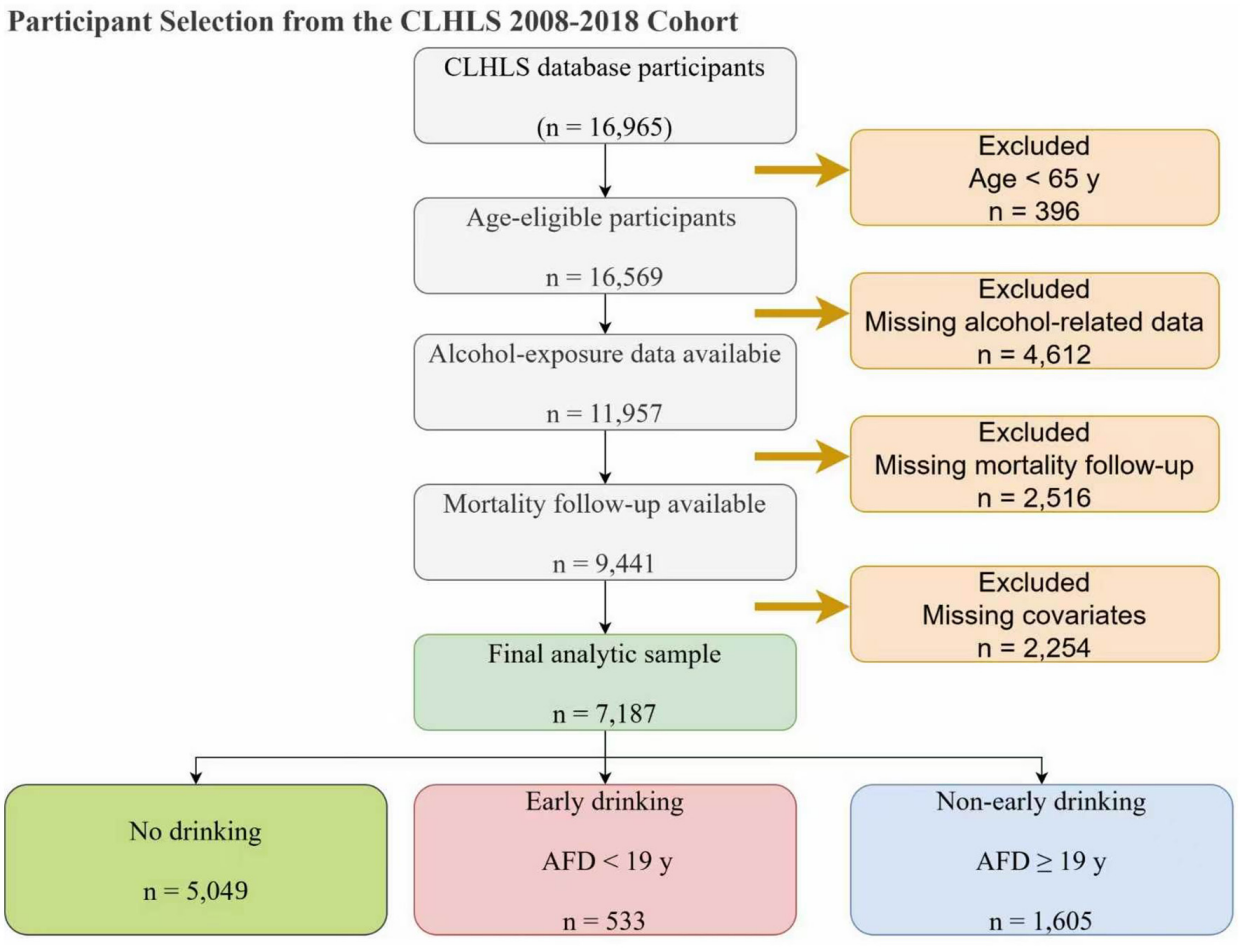
Inclusion Flowchart.

This study initially included 16,965 participants from the CLHLS baseline survey in 2008 (Figure 1). Firstly, 396 individuals under the age of 65 were excluded, leaving 16,569 participants. Then, 4,612 individuals with missing alcohol consumption information were excluded, resulting in 11,957 participants. Subsequently, 2,516 individuals with missing death follow-up data were excluded, leaving 9,441 participants. Finally, 2,254 participants with more than 5% missing main covariates (age, sex, smoking status, physical activity, dietary data, etc.) were excluded, and 7,187 eligible elderly individuals were finally included for analysis. Among them, there were 5,049 individuals who had never drunk alcohol throughout their lives (No drinking), 533 individuals who started drinking early (Early drinking, with the age of first drinking < 19 years), and 1,605 individuals who did not start drinking early (None Early drinking, with the age of first drinking ≥ 19 years). During the average follow-up period of 7.25 years, 3,362 death events occurred.

Table 1 presents the baseline characteristics of the study population, stratified by drinking type (Early drinking, *N* = 533; No drinking, *N* = 5,049; None Early drinking, *N* = 1,605). Significant differences (*p* < 0.05) were observed across groups for several characteristics. The mean age was 81 ± 9, 82 ± 10, and 80 ± 10 years for the Early drinking, No drinking, and None Early drinking groups, respectively (*p* < 0.001). The distribution of sex differed markedly, with the Early drinking group comprising 84.80% males compared to 33.39% in the No drinking group and 78.75% in the None Early drinking group (*p* < 0.001). Significant differences were also noted for ethnicity (*p* = 0.009), educational attainment (*p* < 0.001), self-reported sleep quality (p < 0.001), and the prevalence of smoking (*p* < 0.001), regular exercise (*p* = 0.007), heart disease (*p* = 0.027), and CVD (*p* = 0.006). In contrast, no statistically significant differences were found for BMI (*p* = 0.063), sleep hours (*p* = 0.172), income (*p* = 0.184), hPDI score (*p* = 0.063), or the prevalence of hypertension (*p* = 0.107), diabetes (*p* = 0.190), cancer (*p* = 0.978), or dementia (*p* = 0.136).

**Table 1:** Participants demographics and baseline characteristics.

Characteristic	Drinking type			*p*-value
	Early drinking N = 533	No drinking *N* = 5,049	None early drinking *N* = 1,605	
**Age**				< 0.001^a^
Mean ± SD	81 ± 9	82 ± 10	80 ± 10	
Median (Q1, Q3)	81 (73, 88)	82 (73, 90)	80 (71, 88)	
**Sex**, ***n*** **(%)**				< 0.001^b^
Female	81 (15.20%)	3,363 (66.61%)	341 (21.25%)	
Male	452 (84.80%)	1,686 (33.39%)	1,264 (78.75%)	
**BMI**, ***n*** **(%)**				0.063^b^
< 25	468 (87.80%)	4,264 (84.45%)	1,331 (82.93%)	
>29.9	17 (3.19%)	186 (3.68%)	56 (3.49%)	
25 to 29.9	48 (9.01%)	599 (11.86%)	218 (13.58%)	
**Smoking**, ***n*** **(%)**	383 (71.86%)	1,041 (20.62%)	1,036 (64.55%)	< 0.001^b^
**Ethnic**, ***n*** **(%)**				0.009^b^
Han	517 (97.00%)	4,731 (93.70%)	1,510 (94.08%)	
Others	16 (3.00%)	318 (6.30%)	95 (5.92%)	
**Marital**, ***n*** **(%)**				
Currently married and living with spouse	290 (54.41%)	2,045 (40.50%)	872 (54.33%)	
Divorced	2 (0.38%)	14 (0.28%)	4 (0.25%)	
Never married	8 (1.50%)	32 (0.63%)	27 (1.68%)	
Separated	15 (2.81%)	88 (1.74%)	45 (2.80%)	
Widowed	218 (40.90%)	2,870 (56.84%)	657 (40.93%)	
**Education**, ***n*** **(%)**				< 0.001^b^
Educated	282 (52.91%)	1,851 (36.66%)	920 (57.32%)	
No education	251 (47.09%)	3,198 (63.34%)	685 (42.68%)	
**Sleep quality**, ***n*** **(%)**				< 0.001^b^
Bad	54 (10.13%)	664 (13.15%)	177 (11.03%)	
Good	244 (45.78%)	2,044 (40.48%)	732 (45.61%)	
so so	115 (21.58%)	1,330 (26.34%)	322 (20.06%)	
Very bad	16 (3.00%)	74 (1.47%)	23 (1.43%)	
Very good	104 (19.51%)	937 (18.56%)	351 (21.87%)	
**Sleep hours**				0.172^a^
Mean ± SD	7.87 ± 2.48	7.84 ± 2.63	7.92 ± 2.42	
Median (Q1, Q3)	8.00 (6.00, 10.00)	8.00 (6.00, 10.00)	8.00 (6.00, 10.00)	
**Income**				0.184^a^
Mean ± SD	28,375 ± 29,647	30,920 ± 30,655	29,899 ± 29,741	
Median (Q1, Q3)	20,000 (5,000, 40,000)	20,000 (6,000, 50,000)	20,000 (6,000, 45,000)	
**Occupation**, ***n*** **(%)**				
Agriculture, forestry, animal husbandry or fishery worker	375 (70.36%)	3,493 (69.18%)	1,025 (63.86%)	
Commercial, service or industrial worker	69 (12.95%)	617 (12.22%)	270 (16.82%)	
Governmental, institutional or managerial personnel	21 (3.94%)	149 (2.95%)	92 (5.73%)	
House worker	15 (2.81%)	352 (6.97%)	37 (2.31%)	
Military personnel	9 (1.69%)	28 (0.55%)	20 (1.25%)	
Never worked	1 (0.19%)	31 (0.61%)	3 (0.19%)	
Others	16 (3.00%)	63 (1.25%)	42 (2.62%)	
Professional and technical personnel	13 (2.44%)	229 (4.54%)	85 (5.30%)	
Self-employed	14 (2.63%)	87 (1.72%)	31 (1.93%)	
**Hpdi**				0.063^a^
Mean ± SD	48.2 ± 5.3	47.7 ± 5.5	47.9 ± 5.6	
Median (Q1, Q3)	48.0 (45.0, 52.0)	48.0 (44.0, 51.0)	48.0 (44.0, 52.0)	
**Exercised**, ***n*** **(%)**	217 (40.71%)	1,856 (36.76%)	653 (40.69%)	0.007^b^
**Hypertension**, ***n*** **(%)**	169 (31.71%)	1,553 (30.76%)	452 (28.16%)	0.107^b^
**Diabetes**, ***n*** **(%)**	23 (4.32%)	261 (5.17%)	66 (4.11%)	0.190^b^
**Heart disease**, ***n*** **(%)**	65 (12.20%)	727 (14.40%)	192 (11.96%)	0.027^b^
**CVD**, ***n*** **(%)**	47 (8.82%)	400 (7.92%)	168 (10.47%)	0.006^b^
**Cancer**, ***n*** **(%)**	4 (0.75%)	46 (0.91%)	15 (0.93%)	0.978^c^
**Dementia**, ***n*** **(%)**	19 (3.56%)	172 (3.41%)	39 (2.43%)	0.136^b^

^a^Kruskal–Wallis rank sum test.

^b^Pearson's Chi-squared test.

^c^Fisher's exact test. BMI, body mass index; CVD, cerebrovascular disease; hPDI, healthy plant-based diet index; IQR, interquartile range; SD, standard d.

**Figure 2:**
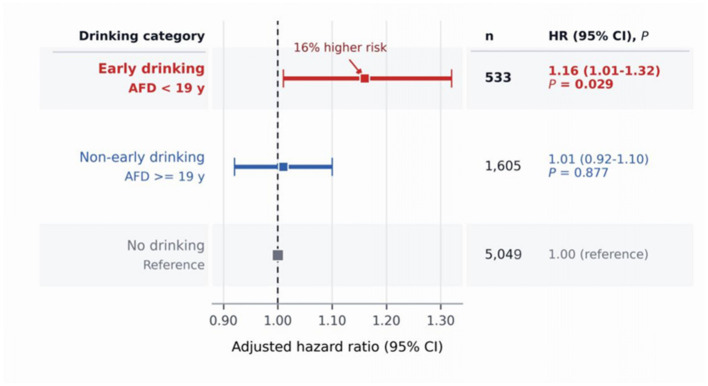
Main analysis results.

The Cox proportional hazards regression model analysis (Table 2) showed that in Model 1 without adjusting for any covariates, the all-cause mortality risk in the early drinking group was 15% higher than that in the never-drinking group (HR = 1.15, 95% CI: 1.02–1.30, *p* = 0.025); there was no significant difference between the non-early drinking group and the never-drinking group (HR = 0.94, 95% CI: 0.87–1.03, *p* = 0.167). In Model 2, which adjusted for sociodemographic, lifestyle, BMI and dietary quality (hPDI), the HR of the early drinking group was 1.14 (95% CI: 1.00–1.29, *p* = 0.052), which was marginally significant. After further adjusting for baseline chronic disease history (Figure 2), the all-cause mortality risk in the early drinking group still significantly increased by 16% (HR = 1.16, 95% CI: 1.01–1.32, *p* = 0.029), while there was no statistical significance in the non-early drinking group (HR = 1.01, 95% CI: 0.92–1.10, *p* = 0.877). In all three models, the trend P value of drinking type and mortality risk was greater than 0.05.

**Table 2:** Cox risk regression analysis of alcohol intake in early life and adulthood and all-cause mortality.

Characteristic	Model 1			Model 2			Model 3		
	HR	95% CI	*p*-value	HR	95% CI	*p*-value	HR	95% CI	*p*-value
Drinking types									
No drinking	—	—		—	—		—	—	
Early drinking	1.15	1.02, 1.30	0.025	1.14	1.00, 1.29	0.052	1.16	1.01, 1.32	0.029
None early drinking	0.94	0.87, 1.03	0.167	0.94	0.86, 1.03	0.208	1.01	0.92, 1.10	0.877
*P* for trend			0.346			0.265			0.744

CI, confidence interval; HR, hazard ratio.

After adjusting for all covariates, the Cox risk regression analysis of alcohol intake in early and later life and all-cause mortality.

Using never drinkers as the reference group, we analyzed early drinkers stratified by daily alcohol consumption ( ≤ 1 cup, 2 cups, ≥3 cups; Table 3). In the fully adjusted model (Model 3), early drinkers consuming ≥3 cups/day had a 34% higher risk of all-cause mortality compared with never drinkers (HR = 1.34, 95% CI: 1.09–1.65, *p* = 0.005), whereas those consuming ≤ 1 cup (HR = 1.00, 95% CI: 0.84–1.19, *p* = 0.978) or 2 cups (HR = 1.22, 95% CI: 0.93–1.59, *p* = 0.145) did not show statistically significant associations. To further examine the dose-response relationship among early drinkers, we then used 1 cup/day as the reference category. In this analysis, early drinkers consuming ≥3 cups/day had a 38% higher risk of all-cause mortality than those consuming ≤ 1 cup/day (HR = 1.38, 95% CI: 1.06–1.79, *p* = 0.015). Moreover, the linear trend test indicated a significant dose–response relationship between increasing daily alcohol intake and higher all-cause mortality risk (*p* for trend < 0.05).

**Table 3:** Dose-response analysis of daily alcohol intake among early drinkers and comparison with never drinkers.

Characteristic		Model 1			Model 2			Model 3			
	N	Event *N*	HR	95% CI	*p*-value	HR	95% CI	*p*-value	HR	95% CI	*p*-value
No drinking vs. Early drinking											
No drinking	5,049	2,348	—	—		—	—		—	—	
Early drinking ( ≤ 1 cup)	284	144	1.01	0.85, 1.19	0.944	1.02	0.86, 1.20	0.857	1.00	0.84, 1.19	0.978
Early drinking (2 cups)	107	57	1.25	0.96, 1.62	0.101	1.23	0.95, 1.60	0.117	1.22	0.93, 1.59	0.145
Early drinking (>= 3 cups)	142	98	1.38	1.13, 1.69	0.002	1.39	1.13, 1.70	0.002	1.34	1.09, 1.65	0.005
Early drinking dose											
Early drinking ( ≤ 1 cup)	284	144	—	—		—	—		—	—	
Early drinking (2 cups)	107	57	1.23	0.90, 1.67	0.192	1.24	0.91, 1.69	0.182	1.23	0.90, 1.69	0.187
Early drinking (>= 3 cups)	142	98	1.40	1.08, 1.81	0.010	1.41	1.09, 1.83	0.010	1.38	1.06, 1.79	0.017
*p* for trend					0.009			0.009			0.015

CI, confidence interval; HR, hazard ratio

Model 1: no covariates were adjusted. Model 2: adjusted for smoking, occupation, income, exercised, bmi, sleep hours, age, sex, ethnic, marital, education, and hPDI. Model 3: adjusted for hypertension, diabetes, heart disease, CVD, cancer, dementia, smoking, occupation, income, exercised, bmi, sleep hours, age, sex, ethnic, marital, education and hPDI.

In age-stratified analyses (Table 4), among participants younger than 75 years, the early drinking group had a 99% higher risk of all-cause mortality than the never-drinking group (HR = 1.99, 95% CI: 1.46–2.69, *p* < 0.001); the non-early drinking group also had a 33% higher risk (HR = 1.33, 95% CI: 1.06–1.68, *p* = 0.015). Among participants aged 75–89 years, no significant association was observed for either the early drinking group (HR = 1.04, 95% CI: 0.87–1.24, *p* = 0.689) or the non-early drinking group (HR = 0.95, 95% CI: 0.83–1.09, *p* = 0.476). Among participants aged ≥90 years, neither group showed a significant association (early drinking group: HR = 1.09, 95% CI: 0.86–1.39, *p* = 0.474). The *p* value for the interaction between age group and drinking type was 0.002.

**Table 4:** Cox risk regression analysis of different alcohol intake patterns based on age stratification and all-cause mortality.

Subgroup	Drinking types	*N*	Adjusted HR (95% CI)^*^	*P* value	*P* for interaction
Overall	No drinking	5,049	Ref		
	Early drinking	533	1.16 (1.02–1.32)	0.026	
	None Early drinking	1,605	1.01 (0.92–1.11)	0.845	
Age group					0.002
< 75	No drinking	1,615	Ref		
	Early drinking	174	1.99 (1.46–2.69)	< 0.001	
	None Early drinking	632	1.33 (1.06–1.68)	0.015	
>89	No drinking	1,356	Ref		
	Early drinking	99	1.09 (0.86–1.39)	0.474	
	None Early drinking	309	1.02 (0.88–1.20)	0.768	
75 to 89	No drinking	2,076	Ref		
	Early drinking	260	1.04 (0.87–1.24)	0.689	
	None Early drinking	664	0.95 (0.83–1.09)	0.476	

^*^Adjusted for hypertension, diabetes, heart disease, CVD, cancer, dementia, smoking, occupation, income, exercised, bmi, sleep hours, sex, ethnic, marital, education and hPDI.

### 3.1 Subgroup analysis results

To evaluate the consistency of the association between early drinking and all-cause mortality risk across different populations, we conducted subgroup analyses based on sex, BMI, hypertension, diabetes, heart disease, cerebrovascular disease, cancer, dementia, smoking status, occupation, physical activity, sleep quality, ethnicity, marital status, and education level. The interaction test showed that the history of heart disease (*p* for interaction = 0.040), history of dementia (*p* for interaction = 0.031), and marital status (*p* for interaction = 0.012) had significant effect-modifying effects on the association between early drinking and mortality risk (Supplementary Table 1).

The subgroup analysis revealed that the association between early alcohol consumption and the risk of all-cause mortality varied among different populations. Regarding sex, the mortality risk of male early drinkers showed a marginal increase (HR = 1.15, *p* = 0.052), while no significant association was observed in females (interaction *p* = 0.337). In the BMI subgroup, only those with overweight (25–29.9 kg/m^2^) had a significantly increased risk by 68% (HR = 1.68, *p* = 0.016), while there was no significant association in the BMI < 25 and >29.9 groups (interaction *p* = 0.220). It is notable that both history of heart disease and history of dementia showed significant effect modification (interaction *p* was 0.040–0.031 respectively): those with a history of heart disease had an increased risk of early alcohol consumption by 63% (HR = 1.63, *p* = 0.004), while those without a history of heart disease showed no significant association (HR = 1.10, *p* = 0.160); those with a history of dementia had an increased risk by 91% (HR = 1.91, *P* = 0.015), and those without dementia also had an increased risk of 14% (HR = 1.14, *p* = 0.040), but the effect was weaker. Marital status also had a significant modifying effect (interaction *p* = 0.012): in married and living together individuals, the risk of early alcohol consumption increased by 60% (HR = 1.60, *p* < 0.001), while the risk in the non-early drinking group also increased by 16% (HR = 1.16, *p* = 0.037); however, no significant association was observed in those who were widowed, separated, divorced or unmarried (the sample size of some subgroups was small, and the results should be interpreted with caution). Regarding education level, regardless of whether they had received education or not, the mortality risk of early drinkers increased by approximately 23%−24% (interaction *p* = 0.261). Additionally, in subgroups such as hypertension, diabetes, cerebrovascular disease, cancer, smoking, physical activity, sleep quality, ethnicity and occupation, no significant association between early alcohol consumption and the risk of death was observed (all interaction *p* > 0.05).

### 3.2 Sensitivity analyses

We performed three sensitivity analyses (presented in Supplementary Tables 2–4). First, we excluded participants with baseline chronic diseases (hypertension, diabetes, heart disease, coronary heart disease, dementia, and cancer), resulting in 4,095 participants, of whom 303 were early drinkers. We then repeated the main analysis. After adjusting for covariates, early drinking remained positively associated with all-cause mortality (HR = 1.19, 95% CI: 1.01–1.41, *p* = 0.043). Second, we excluded participants who died within the first 2 years of follow-up and repeated the main analysis. The results remained consistent, with early drinking showing a positive association with all-cause mortality (HR = 1.14, 95% CI: 1.01–1.30, *p* = 0.037). Third, we performed propensity score matching using age, sex, BMI, and smoking status as matching variables. After matching, the standardized mean differences (SMD) for all covariates were < 0.1. We then repeated the main analysis, which confirmed a positive association between early drinking and all-cause mortality (HR = 1.19, 95% CI: 1.01–1.41, *p* = 0.049).

### 3.3 Missing data comparison and multiple imputation analysis

As shown in Supplementary Tables 5–6, participants with missing alcohol-exposure information differed from those included in the complete-case analysis in several baseline characteristics. Compared with included participants, those with missing alcohol-exposure information were older, more likely to be female and widowed, less likely to smoke or exercise regularly, and had longer sleep duration. These differences suggested that complete-case analysis might introduce selection bias.

We therefore performed an additional multiple imputation sensitivity analysis and repeated the Cox proportional hazards regression model. In the imputed analysis, 8,408 participants were classified as never drinkers, 830 as early drinkers, and 2,561 as non-early drinkers. The results remained consistent with the complete-case analysis. Compared with never drinkers, early drinking was still associated with a higher risk of all-cause mortality (HR = 1.142, 95% CI: 1.037–1.257, *p* = 0.007), whereas non-early drinking was not significantly associated with all-cause mortality (HR = 0.975, 95% CI: 0.915–1.040, *p* = 0.446). These findings supported the robustness of the main results despite missing exposure data.

## 4 Discussion

A total of 7,187 community-dwelling elderly individuals aged 65 and above were systematically evaluated for the long-term association between the age of first drinking (AFD) and all-cause mortality. The results showed that compared with those who had never drunk alcohol, the risk of all-cause mortality was significantly increased by 16% for those who began drinking early (AFD < 19 years old) (HR = 1.16, 95% CI: 1.01–1.32); however, there was no significant increase in mortality risk for those who began drinking later (AFD ≥ 19 years old). The dose-response analysis further indicated that early drinkers with a daily alcohol intake of ≥ 3 cups had a significantly increased mortality risk by 38% (HR = 1.38, 95% CI: 1.06–1.79, *p* = 0.015), and a linear trend was observed (*p* for trend < 0.001). Subgroup analysis revealed that the adverse effect of early drinking on mortality risk was more prominent in individuals under 75 years old (HR = 1.99), those with a history of heart disease (HR = 1.63), those with a history of dementia (HR = 1.91), and those who were married and lived with their spouses (HR = 1.60). Sensitivity analyses (excluding patients with chronic diseases, excluding early deaths, propensity score matching) all supported the robustness of the main results.

The findings of this study are largely consistent with those from previous studies conducted in high-income Western countries. For instance, the epidemiological catchment area study in the United States, based on a 27-year follow-up, revealed that the risk of all-cause mortality was significantly elevated among those who had their first intoxication before the age of 15 (HR: 1.47, 95% CI: 1.25, 1.72) (8); the Finnish birth cohort study also showed that starting to drink alcohol before the age of 18 was associated with an increased risk of premature death before the age of 30 (10). Similarly, a study from South Korea showed that compared with lifelong non-drinkers, middle-aged Koreans with an age of first drinking (AFD) under 19 years had a significantly increased risk of all-cause mortality by 29% (HR = 1.29, 95% CI: 1.02–1.64). While the risk for those with an AFD of 19 years or older increased (by 15%), it did not reach statistical significance (15). Compared with these studies, this research is the first to confirm in the elderly population of China the independent harm of early alcohol consumption on long-term survival, and has further extended the observation window to the elderly age group. It is worth noting that although the overall mortality risk in the non-early drinking group did not significantly increase in this study, in the < 75-year-old subgroup, the risk of death still increased by 33% (HR = 1.33), suggesting that even starting to drink alcohol after adulthood, the long-term cumulative effect may still have an adverse impact on relatively young elderly people. This finding is consistent with the research results of Millwood et al. in Chinese adults (16).

### 4.1 The potential mechanisms underlying the increased risk of death due to early alcohol consumption

Adolescence and young adulthood represent sensitive windows for the development of key neural circuits, such as the prefrontal cortex and limbic system. Earlier alcohol exposure may induce neuroadaptive changes that enhance the rewarding effects of alcohol, thereby leading to higher alcohol consumption and more frequent intoxication behaviors later in life (7, 17, 18). Moreover, early drinkers often exhibit other health-risk behaviors (e.g., smoking, unhealthy diet, and insufficient physical activity). Even after multivariable adjustment in this study, these behaviors did not fully account for the observed associations, suggesting that early alcohol consumption itself exerts a direct toxic effect on physiological systems.

Notably, the association between early drinking and all-cause mortality was strongest among participants younger than 75 years (HR = 1.99), whereas the HRs were attenuated among those aged 75–89 years (HR = 1.04) and ≥90 years (HR = 1.09). This pattern requires careful interpretation given the study design. The CLHLS cohort enrolled only community-dwelling adults aged 65 years and older at baseline. Consequently, participants had to survive from their drinking initiation (which could be as early as adolescence) to at least age 65 to be included in the analysis. This left-truncated cohort is therefore composed of “survivors” who have already lived for decades after their first drink (19). Individuals who were most susceptible to the long-term adverse effects of early drinking—such as those who developed alcohol-related cirrhosis, cardiomyopathy, cancer, or fatal injuries at younger ages—likely died before reaching age 65 and were never included in the study (20, 21). As a result, the attenuated HRs in the older age groups (75–89 years and ≥90 years) should not be interpreted as evidence that early drinking is harmless among the very old. Instead, they likely reflect healthy survivor bias (or depletion of susceptible individuals), whereby the most vulnerable early drinkers have already been selected out of the risk set, leaving a healthier subpopulation with greater resilience to alcohol's toxic effects. This survivor bias would systematically attenuate the true hazard ratio toward the null in the oldest age groups. Therefore, the strong association observed in the younger-old subgroup (< 75 years) likely provides a less biased estimate of the true effect of early drinking on mortality, whereas the null associations in the older subgroups are probably underestimates due to selective survival. In addition, competing risks (e.g., frailty, infections, organ failure) may overshadow the contribution of alcohol in the oldest old, and drinking behavior may have changed over time (e.g., reduction or cessation due to health problems), further complicating the interpretation (22, 23). Taken together, these findings suggest that interventions targeting early drinking among younger older adults (aged 65–74 years) may yield the greatest survival benefits, but the absence of an association in the oldest old should not be taken as evidence of safety. Furthermore, the association between early drinking and all-cause mortality was significantly stronger among older adults with a history of heart disease (*P* for interaction = 0.040) or dementia (p for interaction = 0.031). Potential mechanisms include direct myocardial damage and alcoholic cardiomyopathy induced by alcohol (24). Additionally, alcohol may increase cardiovascular risk by elevating blood pressure, promoting platelet aggregation, and enhancing oxidative stress (24–26). In individuals with dementia, alcohol may further accelerate cognitive decline, impair medication adherence and self-care ability, and consequently increase mortality risk. Moreover, previous studies have suggested that the long-term neurotoxicity of early alcohol exposure may have a synergistic effect with dementia-related pathologies, such as hippocampal atrophy (27, 28).

With respect to marital status, early drinkers who were married and living with their spouse had a 60% higher risk of mortality compared with never drinkers (HR = 1.60, 95% CI: 1.34–1.92, as shown in the subgroup analysis). In contrast, no significant association was observed among widowed, divorced, or never-married participants. This pattern may be explained by the “social drinking” norm within Chinese culture. Married individuals are more likely to maintain regular drinking during family meals and social gatherings, whereas single or widowed individuals may decrease their alcohol intake owing to fewer social opportunities.

#### 4.1.1 Public health policy and clinical implications

##### 4.1.1.1 Policy implications

Based on the findings, we propose the following policy recommendations, with attention to concrete implementation strategies.

First, delay the age of alcohol initiation through school-based education. Alcohol harm education should begin in upper elementary and junior high school, before most children are exposed to drinking. Curricula should include not only general health harms but also evidence that starting before age 18 independently increases long-term mortality risk, even decades later. To enhance efficacy, such programs should involve parents, use interactive formats (e.g., role-playing refusal skills), and be reinforced across multiple grade levels. In addition, enforcement of the legal drinking age (18 years in China) should be strengthened (29), for example through regular compliance checks at alcohol retailers and penalties for sales to minors.

Second, implement family- and community-based interventions for high-risk adolescents. School-based programs are most effective when combined with family engagement. Community health workers could deliver brief counseling to families with adolescents who have already started drinking, emphasizing that reducing or stopping early drinking may mitigate long-term risk (8). Peer-led interventions, such as student-led awareness campaigns, may also help shift social norms around early drinking.

Third, integrate age-at-first-drink screening into routine health check-ups for older adults. For older adults, clinicians should ask not only about current drinking status but also about the age at which they started drinking. This is particularly important for early drinkers (initiation < 19 years) who have additional risk factors, including heart disease, dementia, or high current alcohol intake (≥3 cups/day). For these individuals, individualized advice on alcohol cessation or reduction should be provided, with follow-up support where feasible (21).

Fourth, emphasize dose reduction for early drinkers who cannot abstain completely. For early drinkers unwilling or unable to stop entirely, it should be clearly communicated that reducing daily intake to ≤ 1 cup (as defined by the participant's usual drinking vessel) is associated with lower mortality risk compared with higher consumption (≥3 cups/day). While the exact gram amount cannot be specified due to data limitations, the key message is that lower intake is preferable.

##### 4.1.1.2 Clinical implications

The findings also have direct implications for clinical practice.

First, age at first drink adds prognostic information beyond current drinking status. Current clinical guidelines for alcohol screening (e.g., AUDIT-C) focus on recent consumption (30). Our results suggest that asking older patients about their age at drinking initiation can help identify a subgroup—early drinkers—who may be at higher mortality risk even if their current intake is moderate. Clinicians could therefore add a single question: “At what age did you first start drinking?” to their routine history-taking for older adults.

Second, early drinkers with coexisting conditions warrant intensified risk reduction. The subgroup analyses showed that early drinking was associated with particularly high mortality risk among patients with heart disease (HR = 1.63) or dementia (HR = 1.91). For these patients, clinicians should prioritize alcohol cessation counseling, monitor for alcohol-related complications, and consider more frequent follow-up. Similarly, early drinkers who are married and living with a spouse had a 60% higher risk (HR = 1.60), possibly due to regular social drinking; this group may benefit from family-based alcohol reduction interventions.

Third, the absence of association in very old age groups should not be interpreted as safety. As discussed in the Limitations, the attenuated HRs among participants aged ≥75 years likely reflect healthy survivor bias rather than true harmlessness. Clinicians should therefore continue to advise against heavy drinking even in older patients, regardless of their age.

Fourth, the dose-response finding supports advising even light drinkers against increasing intake. The linear trend (*P* for trend < 0.001) and the significantly elevated risk at ≥3 cups/day (HR = 1.38) suggest that for early drinkers, any increase in daily intake may raise mortality risk. Clinicians can use this evidence to counsel patients who drink at lower levels that maintaining or reducing-rather than increasing intake is important for long-term survival (31, 32).

### 4.2 Rese**arch advantages and limitations**

This study has the following advantages: Firstly, in terms of the research design, it is based on a national, large-sample (7,187 individuals), long-term follow-up (up to 10 years) longitudinal cohort study (CLHLS), covering 23 provinces in China. The results have good extrapolation capabilities. Unlike previous studies that only focused on young or middle-aged populations, this study is the first to systematically assess the association between the AFD and all-cause mortality in the elderly population in China, and extends the follow-up window to the elderly (some participants are over 100 years old), providing direct evidence for “survivor bias”. Secondly, the exposure definition is clear and has policy transformation value. Early drinking is defined as “starting before the legal drinking age (18 years old)”, which is convenient for public health decision-makers to understand and promote. Thirdly, the covariate control is adequate. Not only conventional factors such as social demographics, lifestyle, and BMI are included, but also the hPDI, a cutting-edge dietary quality indicator, is adopted, significantly enhancing the comprehensiveness of confounding control. Fourthly, the statistical strategy is rigorous. Three progressive Cox models (coarse model → social demographics + lifestyle → further correction for chronic disease history) are constructed, and Schoenfeld residuals are used to verify the proportional hazards assumption. Fifth, multiple sensitivity analyses cross-validate, including excluding patients with baseline chronic diseases, excluding those who died within 2 years before follow-up (reducing reverse causality bias), and propensity score matching (PSM), all consistently support the main conclusion (HR 1.19–1.33, all *p* < 0.05), indicating that the results are not easily influenced by confounding bias or reverse causation.

This study has several limitations. First, age at first drinking was retrospectively self-reported. The mean baseline age of our cohort was approximately 81 years, meaning that many participants were asked to recall drinking initiation that may have occurred 60 or more years earlier (i.e., during adolescence or young adulthood). Such long-term recall is prone to substantial misclassification, including inaccurate reporting of exact age at first drink and potential omission of early drinking episodes. If this misclassification were non-differential with respect to mortality, it would likely bias the hazard ratios toward the null, leading to an underestimation of the true association between early drinking and all-cause mortality. Differential recall (e.g., healthier participants being more or less likely to accurately report early drinking) cannot be fully excluded. Second, the exposure did not differentiate regular drinking from occasional attempts; individuals who drank only once or twice before age 18 were classified as early drinkers. This non-differential misclassification likely attenuates effect estimates toward the null, so the true effect may be underestimated. Third, drinking behavior may have changed over 10 years, but we used only baseline exposure without time-dependent updating, potentially causing exposure misclassification. Sensitivity analysis excluding early deaths partially alleviated this concern. Fourth, alcohol intake was assessed using the original CLHLS survey unit (‘cup'). This unit refers to the participant's usual drinking vessel (e.g., a small cup or glass) as commonly used in daily life in China, not an eight-fluid-ounce measuring cup or an internationally standardized drink. Because the survey did not collect information on beverage type (e.g., baijiu, beer, wine) or alcohol concentration (percentage of ethanol), we could not convert the reported intake into grams of pure ethanol or other standardized metrics. Moreover, the dataset did not consistently provide drinking frequency, which prevented us from calculating average weekly consumption. Consequently, light or intermittent drinkers consuming less than 1 cup per day on average were necessarily grouped with those consuming exactly 1 cup per day, and dose-response relationships could not be examined using continuous measures of ethanol intake. These limitations should be considered when interpreting our findings. Fifth, the female early-drinking subgroup was small (*n* = 81), which limited statistical power for sex-specific analyses. Therefore, the null findings among women should be interpreted cautiously, and future studies with larger samples of female early drinkers are needed to confirm our observations. Sixth, beverage types (e.g., baijiu, beer, wine) were not distinguished; they differ in ethanol absorption and toxicity. Chinese older adults predominantly consume high-proof spirits (baijiu), whose hazards may differ from low-proof beverages. Seventh, genetic susceptibility (e.g., ALDH2^*^2) was not accounted for, preventing assessment of gene–environment interactions. Eighth, the study population was limited to community-dwelling older adults; findings may not fully generalize to long-term care facilities or hospitalized patients. Ninth, unmeasured confounders (adolescent family environment, peer pressure, depressive symptoms, socioeconomic status) may remain, potentially influencing both age at first drink and survival in later life. Nevertheless, our three statistical models incorporated many available confounders and effect estimates remained stable across models, supporting robustness; Although the multiple imputation analysis supported the robustness of the main findings, residual selection bias and information bias cannot be completely excluded because missingness may not be fully random and some unmeasured factors related to drinking history may not have been captured. Finally, this study is observational. Despite rigorous prospective design, multi-dimensional adjustment, and multiple sensitivity analyses (e.g., propensity score matching, exclusion of early deaths), residual or unmeasured confounding cannot be completely excluded. Thus, causality between early drinking and all-cause mortality cannot be directly inferred.

## 5 Conclusion

This study indicates that among the elderly population in China, the age at first drinking < 19 years is significantly associated with an increased risk of all-cause mortality, especially for those under 75 years old, with pre-existing heart disease or dementia, who are married and living with their spouses. Delaying the initiation of alcohol consumption should be promoted as an important public health strategy. Future research should further integrate genetic information, types of alcohol consumption and biomarkers to clarify the molecular mechanism by which early alcohol consumption affects long-term survival.

## Data Availability

The original contributions presented in the study are included in the article/Supplementary material, further inquiries can be directed to the corresponding author.
