# Supplementary material for: Age at first drink below 19 years and all-cause mortality in Chinese older adults: a 10-year prospective cohort study
Source: Front Nutr. 2026 Jul 30;13:1873121. doi: 10.3389/fnut.2026.1873121 (PMC13467826; doi:10.3389/fnut.2026.1873121)
Supplement: Supplementary file 1 [file Table_1.docx]

**sTable 1: Subgroup Analysis (Univariate Cox Regression)**

| Subgroup | Drinking.type Levels | N | Adjusted HR (95% CI) | P value | P for interaction |
| --- | --- | --- | --- | --- | --- |
| Overall | No drinking | 5049 | Ref |  |  |
|  | Early drinking | 533 | 1.15 (1.02-1.30) | 0.025 |  |
|  | None Early drinking | 1605 | 0.94 (0.87-1.03) | 0.167 |  |
| Sex |  |  |  |  | 0.337 |
| female | No drinking | 3363 | Ref |  |  |
|  | Early drinking | 81 | 1.04 (0.77-1.41) | 0.804 |  |
|  | None Early drinking | 341 | 1.04 (0.88-1.22) | 0.681 |  |
| male | No drinking | 1686 | Ref |  |  |
|  | Early drinking | 452 | 1.15 (1.00-1.32) | 0.052 |  |
|  | None Early drinking | 1264 | 0.90 (0.81-1.01) | 0.07 |  |
| BMI |  |  |  |  | 0.220 |
| ˂25 | No drinking | 4264 | Ref |  |  |
|  | Early drinking | 468 | 1.09 (0.96-1.24) | 0.192 |  |
|  | None Early drinking | 1331 | 0.93 (0.85-1.01) | 0.091 |  |
| ˃29.9 | No drinking | 186 | Ref |  |  |
|  | Early drinking | 17 | 1.29 (0.62-2.69) | 0.496 |  |
|  | None Early drinking | 56 | 1.30 (0.82-2.07) | 0.267 |  |
| 25 to 29.9 | No drinking | 599 | Ref |  |  |
|  | Early drinking | 48 | 1.68 (1.10-2.55) | 0.016 |  |
|  | None Early drinking | 218 | 1.06 (0.80-1.39) | 0.694 |  |
| Hypertension |  |  |  |  | 0.529 |
| no | No drinking | 3496 | Ref |  |  |
|  | Early drinking | 364 | 1.12 (0.97-1.30) | 0.121 |  |
|  | None Early drinking | 1153 | 0.91 (0.83-1.01) | 0.074 |  |
| yes | No drinking | 1553 | Ref |  |  |
|  | Early drinking | 169 | 1.20 (0.96-1.49) | 0.111 |  |
|  | None Early drinking | 452 | 1.00 (0.86-1.18) | 0.962 |  |
| Diatebes |  |  |  |  | 0.523 |
| no | No drinking | 4788 | Ref |  |  |
|  | Early drinking | 510 | 1.13 (1.00-1.28) | 0.05 |  |
|  | None Early drinking | 1539 | 0.94 (0.86-1.02) | 0.131 |  |
| yes | No drinking | 261 | Ref |  |  |
|  | Early drinking | 23 | 1.61 (0.95-2.73) | 0.078 |  |
|  | None Early drinking | 66 | 1.08 (0.72-1.61) | 0.722 |  |
| Heart.diease |  |  |  |  | 0.040 |
| no | No drinking | 4322 | Ref |  |  |
|  | Early drinking | 468 | 1.10 (0.96-1.25) | 0.16 |  |
|  | None Early drinking | 1413 | 0.96 (0.88-1.05) | 0.338 |  |
| yes | No drinking | 727 | Ref |  |  |
|  | Early drinking | 65 | 1.63 (1.17-2.27) | 0.004 |  |
|  | None Early drinking | 192 | 0.83 (0.65-1.07) | 0.154 |  |
| CVD |  |  |  |  | 0.574 |
| no | No drinking | 4649 | Ref |  |  |
|  | Early drinking | 486 | 1.13 (1.00-1.28) | 0.058 |  |
|  | None Early drinking | 1437 | 0.93 (0.85-1.01) | 0.092 |  |
| yes | No drinking | 400 | Ref |  |  |
|  | Early drinking | 47 | 1.35 (0.91-2.00) | 0.142 |  |
|  | None Early drinking | 168 | 1.03 (0.80-1.33) | 0.827 |  |
| Cancer |  |  |  |  | 0.069 |
| no | No drinking | 5003 | Ref |  |  |
|  | Early drinking | 529 | 1.14 (1.01-1.29) | 0.029 |  |
|  | None Early drinking | 1590 | 0.94 (0.86-1.02) | 0.12 |  |
| yes | No drinking | 46 | Ref |  |  |
|  | Early drinking | 4 | 2.19 (0.66-7.32) | 0.201 |  |
|  | None Early drinking | 15 | 1.87 (0.93-3.75) | 0.077 |  |
| Dementia |  |  |  |  | 0.031 |
| no | No drinking | 4877 | Ref |  |  |
|  | Early drinking | 514 | 1.14 (1.01-1.29) | 0.04 |  |
|  | None Early drinking | 1566 | 0.94 (0.87-1.03) | 0.196 |  |
| yes | No drinking | 172 | Ref |  |  |
|  | Early drinking | 19 | 1.91 (1.13-3.23) | 0.015 |  |
|  | None Early drinking | 39 | 1.40 (0.95-2.07) | 0.089 |  |
| Smoking |  |  |  |  | 0.623 |
| no | No drinking | 4008 | Ref |  |  |
|  | Early drinking | 150 | 1.23 (0.99-1.54) | 0.065 |  |
|  | None Early drinking | 569 | 0.95 (0.83-1.08) | 0.422 |  |
| yes | No drinking | 1041 | Ref |  |  |
|  | Early drinking | 383 | 1.09 (0.93-1.28) | 0.296 |  |
|  | None Early drinking | 1036 | 0.90 (0.80-1.02) | 0.113 |  |
| Occupation |  |  |  |  | 0.384 |
| agriculture,forestry,animal husbandry or fishery worker | No drinking | 3493 | Ref |  |  |
|  | Early drinking | 375 | 1.16 (1.01-1.34) | 0.035 |  |
|  | None Early drinking | 1025 | 0.93 (0.84-1.03) | 0.147 |  |
| commercial, service or industrial worker | No drinking | 617 | Ref |  |  |
|  | Early drinking | 69 | 1.41 (0.99-2.03) | 0.06 |  |
|  | None Early drinking | 270 | 1.10 (0.87-1.39) | 0.411 |  |
| governmental, institutional or managerial personnel | No drinking | 149 | Ref |  |  |
|  | Early drinking | 21 | 1.07 (0.54-2.12) | 0.843 |  |
|  | None Early drinking | 92 | 0.86 (0.55-1.35) | 0.513 |  |
| houseworker | No drinking | 352 | Ref |  |  |
|  | Early drinking | 15 | 1.07 (0.55-2.08) | 0.851 |  |
|  | None Early drinking | 37 | 0.96 (0.60-1.52) | 0.857 |  |
| military personnel | No drinking | 28 | Ref |  |  |
|  | Early drinking | 9 | 2.62 (0.96-7.16) | 0.061 |  |
|  | None Early drinking | 20 | 1.66 (0.69-4.04) | 0.26 |  |
| never worked | No drinking | 31 | Ref |  |  |
|  | Early drinking | 1 |  |  |  |
|  | None Early drinking | 3 | 0.72 (0.09-5.51) | 0.75 |  |
| others | No drinking | 63 | Ref |  |  |
|  | Early drinking | 16 | 0.99 (0.48-2.02) | 0.973 |  |
|  | None Early drinking | 42 | 0.51 (0.26-1.00) | 0.049 |  |
| professional and technical personnel | No drinking | 229 | Ref |  |  |
|  | Early drinking | 13 | 0.91 (0.37-2.25) | 0.839 |  |
|  | None Early drinking | 85 | 1.48 (0.99-2.22) | 0.058 |  |
| self-employed | No drinking | 87 | Ref |  |  |
|  | Early drinking | 14 | 0.57 (0.20-1.60) | 0.287 |  |
|  | None Early drinking | 31 | 1.36 (0.78-2.37) | 0.285 |  |
| Exercised |  |  |  |  | 0.833 |
| no | No drinking | 3193 | Ref |  |  |
|  | Early drinking | 316 | 1.16 (1.00-1.35) | 0.055 |  |
|  | None Early drinking | 952 | 0.97 (0.88-1.08) | 0.578 |  |
| yes | No drinking | 1856 | Ref |  |  |
|  | Early drinking | 217 | 1.23 (1.00-1.50) | 0.049 |  |
|  | None Early drinking | 653 | 0.95 (0.81-1.10) | 0.458 |  |
| Sleep.quaility |  |  |  |  | 0.279 |
| bad | No drinking | 664 | Ref |  |  |
|  | Early drinking | 54 | 0.97 (0.67-1.41) | 0.875 |  |
|  | None Early drinking | 177 | 0.93 (0.73-1.19) | 0.576 |  |
| good | No drinking | 2044 | Ref |  |  |
|  | Early drinking | 244 | 1.14 (0.95-1.36) | 0.159 |  |
|  | None Early drinking | 732 | 0.94 (0.83-1.07) | 0.363 |  |
| so so | No drinking | 1330 | Ref |  |  |
|  | Early drinking | 115 | 0.95 (0.72-1.26) | 0.739 |  |
|  | None Early drinking | 322 | 0.98 (0.82-1.17) | 0.786 |  |
| very bad | No drinking | 74 | Ref |  |  |
|  | Early drinking | 16 | 1.84 (0.98-3.44) | 0.056 |  |
|  | None Early drinking | 23 | 1.38 (0.74-2.58) | 0.308 |  |
| very good | No drinking | 937 | Ref |  |  |
|  | Early drinking | 104 | 1.46 (1.13-1.90) | 0.004 |  |
|  | None Early drinking | 351 | 0.91 (0.75-1.11) | 0.343 |  |
| Ethnic |  |  |  |  | 0.961 |
| han | No drinking | 4731 | Ref |  |  |
|  | Early drinking | 517 | 1.15 (1.02-1.30) | 0.026 |  |
|  | None Early drinking | 1510 | 0.94 (0.86-1.03) | 0.165 |  |
| others | No drinking | 318 | Ref |  |  |
|  | Early drinking | 16 | 1.06 (0.57-1.97) | 0.865 |  |
|  | None Early drinking | 95 | 0.98 (0.71-1.35) | 0.901 |  |
| Marital |  |  |  |  | 0.012 |
| currently married and living with spouse | No drinking | 2045 | Ref |  |  |
|  | Early drinking | 290 | 1.60 (1.34-1.92) | <0.001 |  |
|  | None Early drinking | 872 | 1.16 (1.01-1.33) | 0.037 |  |
| divorced | No drinking | 14 | Ref |  |  |
|  | Early drinking | 2 | 0.83 (0.10-6.69) | 0.865 |  |
|  | None Early drinking | 4 | 4.44 (1.14-17.37) | 0.032 |  |
| never married | No drinking | 32 | Ref |  |  |
|  | Early drinking | 8 | 1.26 (0.36-4.35) | 0.72 |  |
|  | None Early drinking | 27 | 0.44 (0.18-1.07) | 0.071 |  |
| separated | No drinking | 88 | Ref |  |  |
|  | Early drinking | 15 | 0.98 (0.46-2.09) | 0.955 |  |
|  | None Early drinking | 45 | 0.85 (0.49-1.45) | 0.54 |  |
| widowed | No drinking | 2870 | Ref |  |  |
|  | Early drinking | 218 | 1.10 (0.93-1.31) | 0.267 |  |
|  | None Early drinking | 657 | 1.02 (0.91-1.14) | 0.735 |  |
| Education |  |  |  |  | 0.261 |
| educated | No drinking | 1851 | Ref |  |  |
|  | Early drinking | 282 | 1.24 (1.03-1.49) | 0.022 |  |
|  | None Early drinking | 920 | 1.11 (0.97-1.26) | 0.117 |  |
| no education | No drinking | 3198 | Ref |  |  |
|  | Early drinking | 251 | 1.23 (1.05-1.45) | 0.012 |  |
|  | None Early drinking | 685 | 0.96 (0.85-1.07) | 0.446 |  |

**sTable 2: Sensitivity Analysis 1 (Excluding cases where chronic diseases were present at baseline)**

| **Characteristic** |  |  | **Model 1** | | | **Model 2** | | | **Model 3** | | |
| --- | --- | --- | --- | --- | --- | --- | --- | --- | --- | --- | --- |
|  | **N** | **Event N** | **HR** | **95% CI** | **p-value** | **HR** | **95% CI** | **p-value** | **HR** | **95% CI** | **p-value** |
| Drinking types |  |  |  |  |  |  |  |  |  |  |  |
| No drinking | 2,851 | 1,341 | — | — |  | — | — |  | — | — |  |
| Early drinking | 303 | 166 | 1.06 | 0.90, 1.24 | 0.493 | 1.17 | 0.99, 1.38 | 0.067 | 1.19 | 1.01, 1.41 | 0.043 |
| None Early drinking | 941 | 421 | 0.94 | 0.84, 1.05 | 0.293 | 1.03 | 0.92, 1.16 | 0.580 | 1.04 | 0.93, 1.17 | 0.509 |
| P for trend |  |  |  |  | 0.372 |  |  | 0.464 |  |  | 0.393 |
| Abbreviations: CI = Confidence Interval, HR = Hazard Ratio | | | | | | | | | | | |
| Model 1 : no covariates were adjusted  Model 2 : adjusted for age, sex.  Model 3 : adjusted for smoking, occupation, income, exercised, bmi, sleep hours, age, sex, ethnic, marital, education and hPDI. | | | | | | | | | | | |

**sTable 3: Sensitivity Analysis 2 (Exclude the participants who died in the previous two years)**

| **Characteristic** |  |  | **Model 1** | | | **Model 2** | | | **Model 3** | | |
| --- | --- | --- | --- | --- | --- | --- | --- | --- | --- | --- | --- |
|  | **N** | **Event N** | **HR** | **95% CI** | **p-value** | **HR** | **95% CI** | **p-value** | **HR** | **95% CI** | **p-value** |
| Drinking types |  |  |  |  |  |  |  |  |  |  |  |
| No drinking | 5,049 | 2,348 | — | — |  | — | — |  | — | — |  |
| Early drinking | 528 | 294 | 1.13 | 1.00, 1.27 | 0.051 | 1.13 | 1.00, 1.28 | 0.047 | 1.14 | 1.01, 1.30 | 0.037 |
| None Early drinking | 1,605 | 715 | 0.94 | 0.87, 1.03 | 0.167 | 0.94 | 0.86, 1.02 | 0.149 | 1.01 | 0.93, 1.11 | 0.754 |
| P for trend |  |  |  |  | 0.321 |  |  | 0.295 |  |  | 0.594 |
| Abbreviations: CI = Confidence Interval, HR = Hazard Ratio | | | | | | | | | | | |
| Model 1 : no covariates were adjusted  Model 2 : adjusted for smoking, occupation, income, exercised, bmi, sleep.hours,age, sex, ethnic, marital, education, and hPDI  Model 3 : adjusted for hypertension, diabetes, heart disease, CVD, cancer, dementia, smoking, occupation, income, exercised, bmi, sleep hours, age, sex, ethnic, marital, education and hPDI | | | | | | | | | | | |

**sTable 4: Sensitivity Analysis 2 (After propensity score matching)**

| **Characteristic** |  |  | **Model 1** | | | **Model 2** | | | **Model 3** | | |
| --- | --- | --- | --- | --- | --- | --- | --- | --- | --- | --- | --- |
|  | **N** | **Event N** | **HR** | **95% CI** | **p-value** | **HR** | **95% CI** | **p-value** | **HR** | **95% CI** | **p-value** |
| Drinking types |  |  |  |  |  |  |  |  |  |  |  |
| No drinking | 528 | 250 | — | — |  | — | — |  | — | — |  |
| Early drinking | 529 | 296 | 1.14 | 0.96, 1.35 | 0.123 | 1.14 | 0.96, 1.36 | 0.132 | 1.19 | 1.01, 1.41 | 0.049 |
| Abbreviations: CI = Confidence Interval, HR = Hazard Ratio | | | | | | | | | | | |
| Model 1 : no covariates were adjusted  Model 2 : adjusted for occupation, income, sleep.hours,ethnic, marital, education, and hPDI  Model 3 : adjusted for hypertension, diabetes, heart disease, CVD, cancer, dementia, occupation, income, sleep hours,ethnic, marital, education and hPDI | | | | | | | | | | | |

**sTable 5: Baseline statistical analysis of participants without drinking consumption data and those included in the study.**

| **Characteristic** | **Group** | | **p-value** |
| --- | --- | --- | --- |
|  | **Missing  N = 4,612** | **Including  N = 7,187** |  |
| **Age, Mean ± SD** | 85 ± 11 | 81 ± 10 | <0.001^1^ |
| **Sex, n (%)** |  |  | <0.001^2^ |
| female | 2,643 (57.31%) | 3,785 (52.66%) |  |
| male | 1,969 (42.69%) | 3,402 (47.34%) |  |
| **BMI, Mean ± SD** | 22.1 ± 12.4 | 22.1 ± 23.5 | 0.846^1^ |
| **Ethnic, n (%)** |  |  | <0.001^2^ |
| han | 4,351 (94.34%) | 6,758 (94.03%) |  |
| others | 261 (5.66%) | 429 (5.97%) |  |
| **Marital, n (%)** |  |  | <0.001^2^ |
| currently married and living with spouse | 1,744 (37.81%) | 3,207 (44.62%) |  |
| divorced | 14 (0.30%) | 20 (0.28%) |  |
| never married | 40 (0.87%) | 67 (0.93%) |  |
| separated | 73 (1.58%) | 148 (2.06%) |  |
| widowed | 2,741 (59.43%) | 3,745 (52.11%) |  |
| **Education, n (%)** |  |  | 0.141^2^ |
| educated | 1,896 (41.11%) | 3,053 (42.48%) |  |
| no education | 2,716 (58.89%) | 4,134 (57.52%) |  |
| **Income, Mean ± SD** | 29,069 ± 29,112 | 30,503 ± 30,384 | 0.011^1^ |
| **Hypertension, n (%)** | 1,325 (28.73%) | 2,174 (30.25%) | 0.078^2^ |
| **Diabetes, n (%)** | 234 (5.07%) | 350 (4.87%) | 0.618^2^ |
| **Heart disease, n (%)** | 652 (14.14%) | 984 (13.69%) | 0.494^2^ |
| **CVD, n (%)** | 422 (9.15%) | 615 (8.56%) | 0.267^2^ |
| **Cancer, n (%)** | 243 (5.27%) | 65 (0.90%) | <0.001^2^ |
| **Dementia, n (%)** | 190(4.12%) | 230(3.20%) | 0.136^2^ |
| **Smoking, n (%)** | 610 (13.23%) | 2,460 (34.23%) | <0.001^2^ |
| **Excercise, n (%)** | 1,527 (33.11%) | 2,726 (37.93%) | <0.001^2^ |
| **Sleep.quality, n (%)** |  |  |  |
| bad | 523 (11.37%) | 895 (12.45%) |  |
| good | 1,880 (40.88%) | 3,020 (42.02%) |  |
| so so | 1,272 (27.65%) | 1,767 (24.59%) |  |
| very bad | 73 (1.59%) | 113 (1.57%) |  |
| very good | 851 (18.50%) | 1,392 (19.37%) |  |
| **Sleep.hour, Mean ± SD** | 8.43 ± 6.42 | 7.86 ± 2.57 | <0.001^1^ |
| ^1^Welch Two Sample t-test | | | |
| ^2^Pearson's Chi-squared test | | | |

**sTable 6: The main analysis including data of drinking populations with missing values (adjusted survival regression analysis of different drinking patterns and all-cause mortality)**

| **Characteristic** | **N** | **Event N** | **HR** | **95% CI** | **p-value** |
| --- | --- | --- | --- | --- | --- |
| **Drinking types (adjusted)** |  |  |  |  |  |
| No drinking | 8,408 | 4,101 | — | — |  |
| Early drinking | 830 | 462 | 1.142 | 1.037, 1.257 | 0.007 |
| None Early drinking | 2,561 | 1,214 | 0.975 | 0.915, 1.040 | 0.446 |

Model : adjusted for hypertension, diabetes, heart disease, CVD, cancer, dementia, smoking, occupation, income, exercised, bmi, sleep hours, age, sex, ethnic, marital, education and hPDI
